# Sleep disorders in patients with a first psychotic episode: a case-control study

**DOI:** 10.1192/j.eurpsy.2023.968

**Published:** 2023-07-19

**Authors:** M. Abdellatif, H. Nefzi, J. Nasri, M. Methni, K. Rania, M. Karoui, F. Ellouze

**Affiliations:** Psychiatry “G” department, Razi Hospital, Manouba, Tunisia

## Abstract

**Introduction:**

Patients with chronic schizophrenia experience significant disturbances in the quality and quantity of their sleep and it had been mainly attributed to severity of symptoms and antipsychotic use. Recent studies suggested that antipsychotic-naïve early course patients with schizophrenia and their non-psychotic first-degree relatives also show altered sleep quality.

**Objectives:**

In this study we aimed to compare sleep parameters in antipsychotic-naive first-episode schizophrenia patients to their healthy siblings and age- and sex-matched healthy controls.

**Methods:**

We conducted a cross-sectional, descriptive case-control study in the Psychiatry « G » department at Razi Hospital, for a period of six months. Our sample consisted of three groups: a group of schizophrenic patients, a group of their healthy siblings and a group of healthy controls. The three groups were matched by age and sex. The Positive and Negative Syndrome Scale (PANSS) was used to assess the severity of symptoms in patients with schizophrenia. The Morningness-Eveningness Questionnaire (MEQr), Epworth Sleepiness Scale (ESS), and Pittsburgh Sleep Quality Index (PSQI) were used in the three groups to assess Circadian preference, daytime sleepiness and sleep quality.

**Results:**

There was no significant difference between the groups regarding the chronotype. Patients had significantly higher daytime sleepiness compared to siblings (p=0.001) and controls (p<0.001). Patients also had poorer quality sleep (PSQI total score) than siblings (p<0.001) and controls (p<0.001), longer sleep latency than siblings (p=0.003) and controls (p<0.001); lower habitual sleep efficiency than siblings (p=0.003) and controls (p<0.001). Siblings had poorer sleep quality (p=0.001), longer sleep latency (p=0.006) and shorter sleep duration (p=0.033) compared to control subjects.

**Conclusions:**

Our results joined those of the literature concerning the significant prevalence of sleep disorders in early psychosis. In addition, the alteration of sleep quality in unaffected siblings compared to healthy controls supports the hypotheses suggested in the literature that sleep disorders may be markers of genetic susceptibility to schizophrenia and serve as a potential endophenotype of the disease.

**Disclosure of Interest:**

None Declared

